# Recurrent stress across life may improve cognitive performance in individual rats, suggesting the induction of resilience

**DOI:** 10.1038/s41398-019-0523-5

**Published:** 2019-08-05

**Authors:** Ravit Hadar, Henriette Edemann-Callesen, Elizabeth Barroeta Hlusicka, Franziska Wieske, Martin Vogel, Lydia Günther, Barbara Vollmayr, Rainer Hellweg, Andreas Heinz, Alexander Garthe, Christine Winter

**Affiliations:** 1 Department of Psychiatry and Psychotherapy, Charité University Medicine Berlin, Campus Mitte, Berlin, Germany; 2 Department of Psychiatry and Psychotherapy, Technische Universität Dresden, Medical Faculty Carl Gustav Carus, Dresden, Germany; 3 International Graduate Program Medical Neurosciences, Charité University Medicine Berlin, Berlin, Germany; 4 Department of Psychiatry and Psychotherapy, Heidelberg University, Central Institute of Mental Health, Medical Faculty Mannheim, Mannheim, Germany; 5 German Center for Neurodegenerative Diseases (DZNE) Dresden, Dresden, Germany

**Keywords:** Long-term memory, Molecular neuroscience

## Abstract

Depressive symptoms are often accompanied by cognitive impairments and recurrent depressive episodes are discussed as a potential risk for dementia. Especially, stressful life events are considered a potent risk factor for depression. Here, we induced recurrent stress-induced depressive episodes over the life span of rats, followed by cognitive assessment in the symptom-free period. Rats exposed to stress-induced depressive episodes learned faster than control rats. A high degree of stress-induced depressive-like behavior early in the paradigm was a predictor of improved cognitive performance, suggesting induction of resilience. Subsequently, exposure to lorazepam prior to stress-induced depressive episodes and cognitive testing in a nonaversive environment prevented the positive effect. This indicates a beneficial effect of the stress-associated situation, with the existence of individual coping abilities. Altogether, stress may in some have a beneficial effect, yet for those individuals unable to tackle these aversive events, consecutive unpleasant episodes may lead to worse cognitive performance later in life.

## Introduction

A history of recurrent depression episodes has been suggested to constitute a risk factor for dementia, such that vulnerability toward dementia has been correlated to the frequency of the depressive episodes, with recurrent episodes increasing the risk^1^. In the clinic, cognitive complaints are often reported by depressed patients during the symptomatic, and also the remitted phase and depression has been suggested as an early manifestation of dementia^1,2^. What remains to be investigated is whether and how experiencing depressive episodes influence cognitive performance later in life, long after symptoms have subsided and thus no direct interaction and overlap are evident.

The experience of being unable to avoid or handle stressful life events is considered a potent risk factor for depression^3–7^, and is implemented in the learned helplessness (LH) paradigm that induces a transient, depressive phenotype in rodents^8^. On a behavioral level, the experience of unescapable stress induces helplessness and subsequent anhedonia-like behavior in the animal, whereas on a neurobiological-level serotonergic dysregulation, decrease in brain-derived neurotrophic factor (BDNF) levels and hippocampal deficits become evident^9–12^. The LH paradigm relies on the observation that the response a stressor elicits in an animal is dependent upon its cognitive appraisal^3^. Clinical findings also suggest that the manifestation of depression following an adverse life event depends on the situation, as well as the individual subjected to it^13^. To this end, the controllability of the aversive situation is a critical factor that determines the induction of a depressive phenotype^14–16^. Previously, studies have shown that exposure to stress in the LH paradigm reduces cognitive performance in some individuals when subjects are tested for the acquisition of an escape response after 24 h^17^.

The course of depression varies between individuals, yet the majority of patients experience recurrence following recovery, with the probability of recurrence increasing with every subsequent episode. On a neurobiological level, recurrent stress-induced depression has been shown to induce hippocampal damage as well as alterations in neurogenesis and long-term potentiation, that may be accountable for the frequent cognitive dysfunctions seen in depression^4,5,18^. However, there is no direct evidence that these alterations indeed persist and thus affect cognitive performance later in life. As such, investigation into how cognitive functions are altered following stress-induced depressive episodes on a life-long trajectory is needed. Given the increase in life expectancy worldwide, insight into the link between recurrent stress-induced depression and cognitive performance is of major importance, as for most a causation between recurrent depression and declined cognitive performance would point to the necessity of adequate early treatment for depression. Thus, the aim of this study was to investigate the impact of recurrent, transient stress-induced depressive symptoms on cognitive performance, when induced across the life span of an aging rat.

## Materials and methods

### Animals and housing

Male Sprague–Dawley rats (8 months of age) (*n* = 78) were used. Animals were housed in a temperature and humidity-controlled vivarium with a 12 h light–dark cycle with food and water ad libitum. All experiments were in accordance with the German Animal Welfare Act and approved by the Landesdirektion Sachsen (24-9168.11-1/2013-63).

### Experimental design

**Fig. 1:**
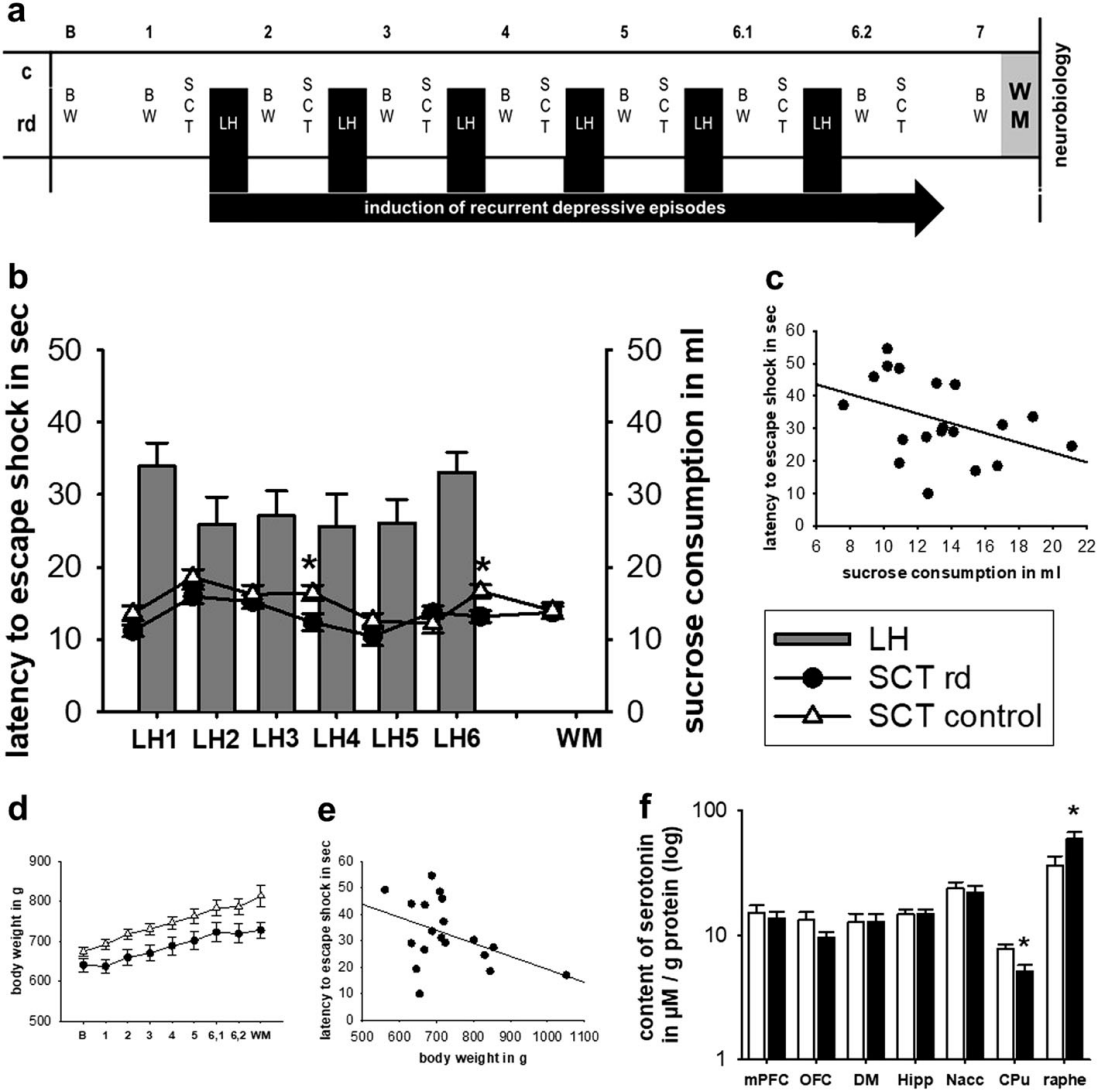
The induction of depressiveness. **a** Experimental design showing the time-points of measurement and induction of recurrent depressive episodes over the life span of an ageing rat. **b** Latency to escape the shock in the LH paradigm and sucrose consumption in the SCT measured over course of the study. **c** Correlation between performance in the LH and SCT. **d** Average body weight in gram over the course of the study. **e** Correlation between latency to escape the shock and body weight in gram. **f** Serotonin levels in various cortical and subcortical areas. LH learned helplessness paradigm, SCT sucrose consumption test, BW body weight, WM water maze, rd recurrent depression, mPFC medial prefrontal cortex, OFC orbitofrontal cortex, DM dorsal medial thalamus, Hipp hippocampus, Nacc nucleus accumbens, CPu caudate putamen, Raphe raphe nucleus. Where applicable, the data are provided either as individual data points or given as mean SEM. Asterisk (*) indicates significant differences between treatment groups

**Fig. 2:**
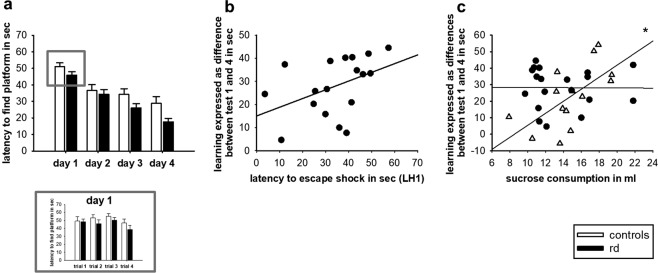
The impact of recurrent depression on cognition. **a** Latency to find the platform in the WM. Performance in the LH paradigm over four consecutive testing days, with the individual four trials presented for the 1st day of testing. **b** Correlation between learning in the WM and performance in the first LH test (LH1). **c** Correlation between performance in the SCT and learning in the WM. rd recurrent depression, LH learned helplessness, WM Water Maze. Where applicable, the data are provided either as individual data points or given as mean SEM. Asterisk (*) indicates significant differences between treatment groups

**Fig. 3:**
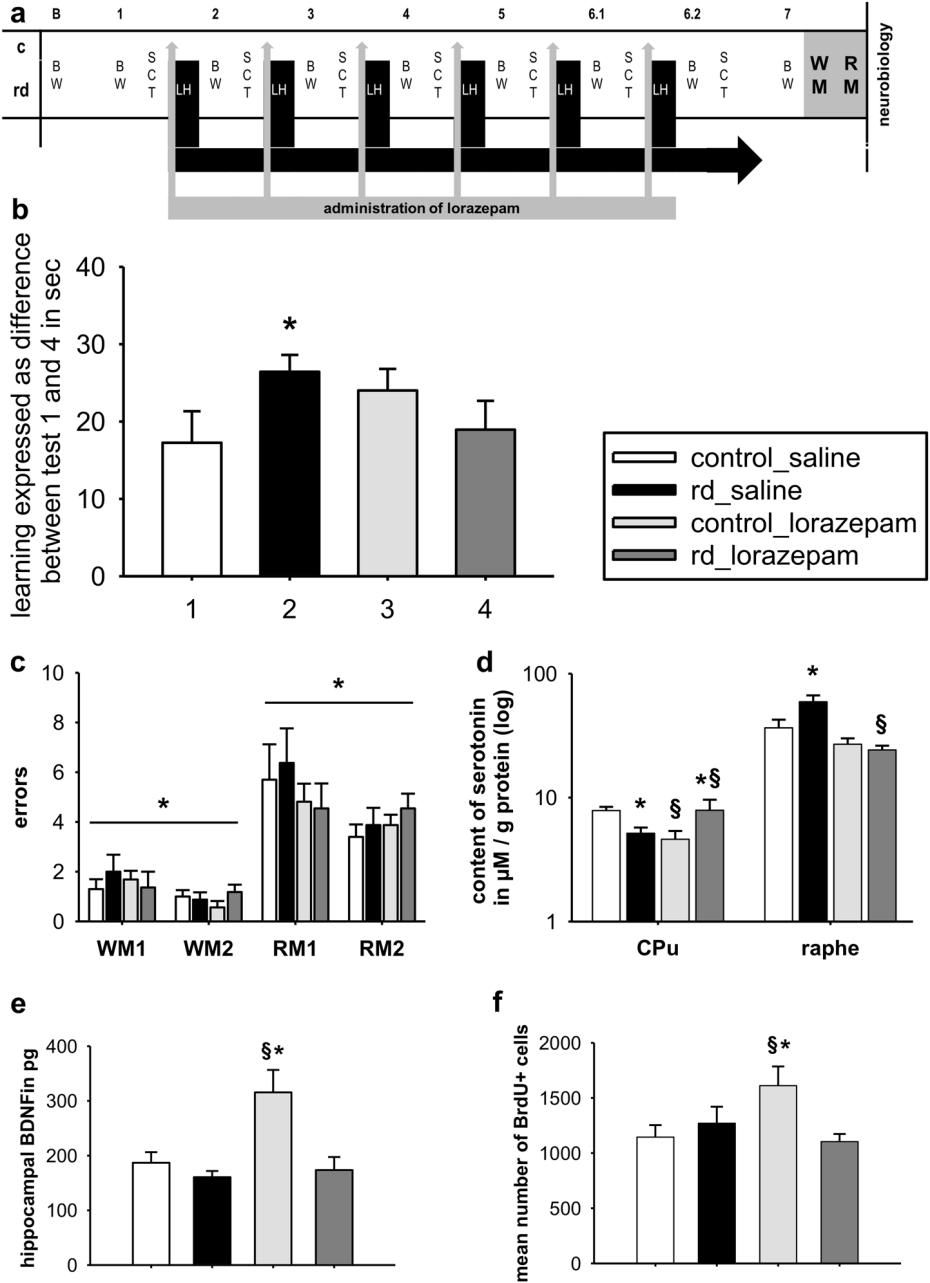
Alleviating the stress and depressiveness. **a** The experimental design showing the time-points for measurement and induction of recurrent depressive episodes alongside administration of lorazepam over the life span of an ageing rat. **b** Learning abilities in the WM for rd and control rats injected with either saline or lorazepam. **c** Errors in reference and working memory at testing day 1 and 2 in the RM (**d**) serotonin levels in the CPu and raphe in rd and control rats injected either with saline or lorazepam (**e**) hippocampal levels of BDNF (**f**) mean number of BrdU+cells. WM water maze, RM paradigm radial maze, WM1/2 working memory test day 1 and 2, RM1/2 reference memory test day 1 and 2, rd recurrent depression, CPu caudate putamen, Raphe raphe nucleus. Where applicable, the data are provided either as individual data points or given as mean SEM. Paragraphs (§) indicate group effects. Asterisk (*) indicates significant differences between treatment groups

In a first set of experiments (*n* = 33), animals were randomly divided into a control group (*n* = 14) and an intervention group (*n* = 19), after which they were followed across a life span for about 10 months. The intervention group was subjected to controlled, transient recurrent depressive (rd) states through the LH paradigm. In addition, the LH paradigm was also used to measure the degree of helplessness present in the individual animal^3,19^. Induction and transiency of depressiveness were assessed through the sucrose consumption test (SCT) which was conducted prior to each LH test session to prove transiency, as well as after the final LH test to verify induction. The SCT was chosen, as it constitutes a stress-free paradigm and thus can be employed to investigate the degree of depressiveness without interfering with symptom presentation. Cognitive performance was later assessed using the Morris water maze (WM). The WM was conducted 6–8 weeks after the last LH test, to prevent the induced stress and depressiveness from having a direct known impact on performance^20^. Control rats went through the same consecutive SCT paradigm, yet did not undergo actual LH testing as they were only placed in the LH conditioning chambers (Fig. 1a). To further assess the impact of the stress mediated by the LH paradigm on cognitive performance, an additional test round was conducted on (*n* = 45). Rats (*n* = 26; 16 controls and 10 rd) were subjected to lorazepam (i.p. 0.3 mg/kg body weight) 30 min prior each LH paradigm and subsequently tested in the WM (Fig. 2a). Rats serving as controls (*n* = 19) received saline injection and underwent the same treatment as above (11 control and 8 rd). In this experiment, all rats were also tested in the radial maze (RM) (Fig. 3a). One week following the last LH test, rats started receiving i.p. injections (50 mg/kg body weight) of BrdU (Sigma-Aldrich, Germany, 0.9% saline) three times/day for 3 days. Following the cognitive paradigm(s), neurobiological investigations were conducted (Fig. 3a).

### Behavioral assessment

**Learned helplessness (LH):** In this study, we employed a well-validated protocol for LH^17^. The setup consisted of an Operant Behavior System Mannheim Type 259900 (TSE, Bad Homburg, Germany) that included an operant conditioning chamber (48.5 × 30 × 21.5 cm) and a shock generator. The LH paradigm took place over two consecutive days. At day 1 (conditioning phase), rats were exposed to an unpredictable, inescapable shock situation for 40 min (200 ms phase duration; intensity of 0.8 mA). Shocks lasted between 5 and 15 s, with an inter-trial time of 5–15 s, leading to a total shock time of 20 min/test. On the consecutive day (test phase), animals were placed in the same conditioning chamber, now including a lever (3.5 × 3.5 cm) and signal light (white, 12 W, 4 cm above the lever). Each shock was indicated by a light clue, and could be terminated through lever press. During the testing phase, animals received 15 shocks of maximum 60 s in duration (inter-trial time: 24 s). Latency to escape the shock (seconds) was calculated for each animal. **Sucrose consumption test (SCT):** The SCT was employed to measure depressiveness^21^. Two days prior to testing, animals were habituated to testing bottles containing water for 30 min. Twenty-four hours prior to testing, animals were habituated to the sweetened condensed milk (Milchmädchen 1:3) for 30 min and food restricted (15 g chow per rat). On testing day, animals were presented with two bottles containing either tap water or sucrose solution for 15 min. Bottles were weighed before and after testing. Sucrose consumption (ml) was calculated relative to body weight. **Water Maze (WM):** The WM was used to assess cognitive performance^22^. The test apparatus consisted of a water-filled (35 cm depth; 22 °C), circular pool (Ø 1,60 m, height: 76 cm) with a submerged (2 cm underwater) hidden platform (15 cm × 15 cm). Visual cues were provided around the pool. Each animal was tested for four trials/day over four consecutive days (60 s acquisition trials, with a retention time on the platform for 5 s). In the first trial, the animal was dropped from the farther quadrant from the platform, and for the next trials from the successive quadrants following a clockwise order. If the animal did not find the platform, then it was guided to it and left sitting for 5 s. Swim paths were recorded via EthoVision Pro 3.1 (Noldus) and latency to find the platform (in seconds) was assessed. Learning was calculated as the time difference (in seconds) to find the platform between the 1st and 4th test. **Radial Arm Maze (RAM):** Animals were food restricted two days prior to testing (20 g chow per rat) and monitored so that there was not a weight loss of more than 20%. The gray plexiglas RAM consisted of an octagonal central platform (47 cm in diameter) elevated 65 cm above the floor, with eight arms (40 cm × 15 cm) radiating from the platform at equal angles, all surrounded by 25 -cm height walls. At the end of each arm, there was a well where the bait was placed. For testing, rats were placed in the center of the RAM. From testing days 1–3, animals freely explored the maze for 10 min or until bait placed in each of the eight arms was consumed. On days 4–8, bait was randomly assigned to four arms doors were closed while placing the rat in the central platform, then opened at the same time and when a rat entered an arm with the whole body it was considered the chosen arm and the doors of the other arms were closed; after eating the bait, the animals were allowed to return to the central platform, the doors were closed again and after a delay of 5 s the doors were open again. Animals explored for 10 min or until all the bait was consumed. The number of correctly entered (baited) arms was annotated, and reference (entry into non-baited arm) and working memory (entry into previously visited arm) was assessed^23^.

### Post mortem neurobiological assessment

Each half of the rats were either transcardially perfused or decapitated following finalization of the WM/RM. After transcardial perfusion, coronal sections of 40 µm for histology and 1 mm for content measurements were cut on a cryostate. After decapitation, brains were extracted and snap-frozen in liquid nitrogen. **Bromodeoxyuridine (BrdU) assessment**: Prepared slices were stained with primary (1:500, rat anti BrdU, AbED Serotec) and secondary antibodies (1:500, donkey anti rat, Dianova). The number of BrdU-positive cells were counted from one-in-6 series of sections (240 μm apart) in the hippocampus (Hipp). **BDNF measurement:** Hippocampal samples were obtained by extracting the whole hippocampus out of three 1- mm slices at bregma level −2.3 to −5.3 according to Paxinos & Watson’s brain atlas. Samples were homogenized by ultrasonication for 20 s in 0.7 ml of extraction buffer containing 100 mM Tris-HCl, 400 mM NaCl, 0.1% NaN_3_, and stored at −80 °C until use. Endogenous hippocampal BDNF concentrations were measured in the rethawed homogenates using commercial ELISA kits in principle according to the manufacturer’s instructions (Promega), but adapted to a highly sensitive fluorometric technique as described in detail elsewhere^24^. BDNF content was expressed as equivalents of recombinant human BDNF. The detection limit of the assay was 1 pg/ml. Determinations of recovery and specific and nonspecific neurotrophin binding (the latter against mouse IgG1 obtained from MOPC 21) involved triplicate fluorescence determinations for each tissue sample. Using this improved fluorometric ELISA, it was feasible to quantify BDNF in brain tissue with a minimal wet weight of ~5 pg^24^. BDNF levels were expressed as picograms per milligram of tissue (wet weight) OR:? g of protein? **High-performance liquid chromatography (HPLC):** Tissue samples were obtained via micropunches of Ø 1 mm from the left medial prefrontal cortex (mPFC, the cingulate cortex area 1, pre- and infralimbic cortex taken together at Bregma level 3.2–2.2, left orbitofrontal cortex (OFC, ventrolateral and lateral part, Bregma 3.2–2.2.), dorsal medial thalamus from both hemispheres (DM, −3.3 to −4.3), left nucleus accumbens (Nacc, 1.7–0.7), left caudate putamen (CPu, 1.7–0.7), and central raphe nucleus (raphe, −7.3 to −8.3). Samples were homogenized immediately by ultrasonication in 250–750 µl 0.1 M perchloric acid at 4 °C. In all, 100 µl of each homogenate was stored at −80 °C for subsequent protein determination. The homogenates were centrifuged at 17.000 *g* and 4 °C for 10 min. Serotonin levels (µM/g protein) were measured in the supernatants via HPLC electrochemical detection.

### Statistics

Behavioral (LH, SCT, WM and RM) and neurobiological (BrdU+, BDNF and serotonin levels) analyses were carried out using a two-way analysis of variance (ANOVA), with testing group and treatment/test days as variables. If applicable, a Holm-sidak post hoc test was performed for multiple comparisons. A two-sample *t* test was applied to assess differences between testing groups. Correlation analyses was performed to assess the association between variables. Significance was set to *p* < 0.05.

## Results

### The induction of transient, depressive episodes by the learned helplessness paradigm

Recurrent depressive episodes were induced through the LH paradigm. Depressiveness was calculated by latency to escape the shock in the LH paradigm, sucrose consumption in the SCT test, body weight, and neurobiological alterations via serotonin content. Latency to escape the shock in the LH paradigm showed that the degree of induced helplessness was stable across consecutive trials. Following LH testing (as shown for the last LH test), rats exposed to recurrent depression (rd-rats) consumed significantly less sucrose solution as compared with control rats (F(1,30) = 8.275, *p* = 0.007) (Fig. 1b). Further assessment indicated a negative correlation between a decrease in sucrose consumption and latency to escape to shock in the last LH test (R = −0.412, *p* = 0.0799) (Fig. 1c). However, this was not evident when assessing sucrose consumption prior to the consecutive LH testing episodes, showing that the prior induced helplessness was transient in nature. Comparing weight gain between the two groups over the experimental course using a two-way ANOVA, showed a significant effect for the factor testing group (F(1,291) = 5.087, *p* = 0.030) and test session (F(8,291) = 127.205, *p* < 0.001) as well as significant interaction (F(8,298) = 2.014, *p* = 0.045), with a further post hoc test indicating that rd-rats generally exhibit lower body weight as compared to control rats (Fig. 1d). Further investigations indicated that a decrease in body weight was negatively correlated with the latency to escape the shock in the last LH test (R = −0.439, *p* = 0.0600) (Fig. 1e). Neurobiological assessment of serotonin content revealed that rd-rats showed an increase in the raphe nucleus (t(30) = −2.193, *p* = 0.036) and a decrease in the CPu as compared with controls (t(31) = 3.192, *p* = 0.003) (Fig. 1f).

### The effect of recurrent depressive episodes on cognitive performance

The impact on cognitive performance was assessed in the WM paradigm. Here, a two-way ANOVA showed a significant effect for the factor testing group (F(1,90) = 6.239, *p* = 0.018) and testing days (F(3,90) = 36.614, *p* < 0.001), with rd-rats generally exhibiting shorter latencies to find the platform as compared with controls. Comparing the two testing groups for performance on the first testing day in the WM paradigm revealed no significant difference (t(30) = 0.135, *p* = 0.894) (Fig. 2a). In addition, investigation into body weight on WM performance found no correlation between body weight and learning abilities in the WM (data not shown).

Interestingly, there was an indication of a positive correlation between LH performance in the first LH test and learning abilities in the WM (t(18) = 0.437, *p* = 0.0701) (Fig. 2b). In rd-rats, learning did not seem to be associated to the hedonic state (t(19) = −0.00860, *p* = 0.972), whereas in controls a positive correlation was found between the hedonic state and learning (t(14) = 0.633, *p* = 0.0151) (Fig. 2c).

### The impact of lorazepam on recurrent depression, cognitive performance, and neurobiological properties

In a two-way ANOVA, a significant interaction was found on performance in the WM following lorazepam (F(1,78) = 4.458, p = 0.038), showing that rd-rats receiving lorazepam did not have improved learning abilities in the WM (Fig. 3b).

Regarding serotonin levels in the raphe nucleus, a two-way ANOVA found a significant effect for the factor treatment (F(1,46) = 9.263, *p* = 0.004), and a significant interaction (F(1,46) = 4.591, *p* = 0.037), with a further post hoc test showing that lorazepam normalized serotonin levels in the rd-rats without affecting the controls rats. A two-way ANOVA found a significant interaction (F(1,47) = 12.085, *p* = 0.001) in the CPu with post hoc tests indicating an increase in serotonin levels in the rd-rats receiving lorazepam, yet a decrease in serotonin levels in the control rats (*p* < 0.05) and (*p* < 0.05). When testing the effect of lorazepam on hippocampal BDNF, a two-way ANOVA showed a significant effect for the factor phenotype (F(1,43) = 13.677, *p* < 0.001) and treatment (F(1,43) = 9.681, *p* = 0.003), as well as a significant interaction (F(1,43) = 6.465, *p* = 0.015). Subsequent post hoc test revealed a significant increase in hippocampal BDNF in the control rats that received lorazepam (*p* < 0.05) (Fig. 3e). In correlation, a two-way ANOVA of the effect of lorazepam on BrDu+cells showed a significant interaction (F(1,78) = 4.458, *p* = 0.038), with a post hoc test revealing an increase in BrDU+cells in the control–lorazepam rats (*p* < 0.05) (Fig. 3f).

### The impact of nonaversive environment on cognitive performance

For both working and reference memory, a two-way ANOVA found a significant effect for testing days (working memory: F(1,41) = 4.189, 0.047; reference memory: F(1,41) = 5.525, *p* = 0.024). For both types of memory, no significant effect was found for the factor testing group or interaction (Fig. 3c).

## Discussion

The aim of this study was to investigate the impact of recurrent stress-induced depression on later cognitive performance. The results show that a history of stress-induced episodes may enhance future learning under stressful conditions, whereas it was not affecting stress-free learning paradigms. In the individual animal, the level of stress experience and depressive-like behavior measured during the first LH episode served as a predictor of the later performance in the cognitive tasks. The results point toward the development of individual coping abilities, for which stress may have a positive impact on cognition.

The induction of depressive-like behavior following LH was evident as rd-rats consumed significantly less sucrose than control rats directly following the final LH test. Depressive-like behavior was also reflected in a decrease in body weight of the rd-rats, as well as upregulated serotonin levels as compared with the control rats. These findings have been previously associated with depressive-like behavior in different animal models^9,25–27^. The correlation between increased serotonin levels and depressive behavior needs to be further investigated. Consumption of sucrose was negatively correlated with the latency to escape the shock, indicating a higher degree of depressive-like behavior in correlation with impaired performance in the LH paradigm

Later assessment of cognitive performance in the symptom-free period showed that rd-rats learned faster than control rats. This was observed in the WM, with the rd-rats displaying a shorter latency to find the platform compared with control rats over consecutive trials. To investigate for confounders, performance in the individual trials at the first testing day of the WM was assessed. Here, rd and control rats performed equivalently, indicating no difference between the two groups at baseline. Further assessment of possible confounders found no correlation between body weight and performance in the WM (data not shown), thus ruling out the impact of body weight on learning in the WM.

However, what seemed to influence the later cognitive performance was the initial severity of symptoms, as high degree of depressive symptom presentation in the first LH testing episode predicted improved learning later in the WM. Surprisingly, this suggests that early depressive episodes may have had a beneficial effect on later cognitive performance in aging rats. In contrast, the hedonic state that in control rats was positively correlated with WM performance, yet this did not have any effect on learning abilities in rd-rats. This might suggest that the clinical observation of an association between improved learning and positive emotions could be abrogated under certain conditions^28^.

These findings raise the question as to whether we have indeed modeled the influence of depression on cognitive performance or rather the impact of resilience?. As such, it may be that the experience of the early aversive LH paradigm has helped the animal to better overcome the later learning task in the also aversive WM, by transferring knowledge from a previous stressful situation to another later stressful event. Indeed, other studies have investigated the correlation between individual resilience and the ability to control stress. Animal studies have found that controllable stress exposure may improve mood and cognitive abilities, which has been linked to an increase in resilience^16,29–31^. Others have found that enhanced learning abilities in rats indicate emotional controllability, which further was linked to gaining resilience^30^. Several studies have shown that the effect of stress on hippocampus-dependent learning, follows an inverted U-shape, in which too low/high stress levels impairs consolidation, whereas moderate stress levels is a prerequisite for long-term memory^32–40^. Fast arrival to the platform in the WM paradigm, as a result of stress, has mainly been observed in animal models, in which specific neurotransmitter systems have been modulated^32,36,41^. Collectively, these studies show that certain neurotransmitter systems broaden the span of attention during stressful condition, in which especially corticosterone plays a facilitating role, as altered corticosterone increases memory storage under stressful conditions. As such, these studies show that an increase in stress levels facilitates neurobiological modulations that leads to better performance in later stressful environments^32,36,41^. As such, stress exposure may in some individuals lead to the development of coping strategies that subsequently moderates the impact of a following stressful challenge and improves adjustment to future stressful settings^31,42^.

Given that we studied resilience in stress-associated situations, one would expect (i) no improvement in cognitive performance if the aversive component from the LH was removed and (ii) no improvement in cognitive performance when assessed in a later nonaversive learning paradigm. To investigate this, we (i) subjected rats to lorazepam directly prior to LH testing, to normalize the aversion induced by the paradigm and (ii) integrated the radial maze to assess learning abilities in a nonaversive environment.

In accordance with our hypothesis, only rats that had been subjected to the aversive LH had better cognitive performance in the WM. Rats injected with lorazepam performed equivalent to control rats. Yet, by removing the stress, we also removed the helplessness for which the experience of a stressor is mandatory. As a result, there no longer was a deregulation of serotonin in the rd-rats after receiving lorazepam, i.e., rats did not display the neurobiological surrogate of depressiveness and consequently no depressive-like behavior^25^. Thus, the question still remains as to whether it is the continuous depressive episodes or rather the stress that impacts cognition later in life. Furthermore, the intensity of recurrent stress episodes tends to variate across a patient’s life span, therefore in order to more accurately mimic the clinical situation, the effect of fluctuating stress levels on coping abilities should be investigated in future studies.

Subsequently, we checked for resilience-associated parameters, such as BrdU cells and BDNF levels, and found an increase of these parameters upon lorazepam administration in controls. Hippocampal BrdU expression and BDNF levels following stress exposure have shown to be regulated in a temporal age-dependent manner, which previously have led to discrepancy when wanting to understand the neurobiological impact of stress on these parameters^43^. The fact that resilience associated parameters are not affected in the rd-rats may support this notion, as the results only reflect the last LH testing episode. However, interestingly we have two different antagonizing effects in controls and rd-rats, suggesting that it is the pathology/vulnerability of the brain that directs the impact of factors that modifies stress.

Learning in the nonaversive RM paradigm, did not differ between rd and control rats in either working or reference memory, showing that improved learning is only found in stress-associated situations. In correlation, studies have shown that especially stress has a differential neurobiological and thus behavioral effect, depending on how the stressor is perceived. Chronic, unpredictable stress induces a decline in hippocampal neurogenesis and through this impairs hippocampal-dependent cognition^5,44–46^. In contrast, mild-to-moderate predictable stressors increase hippocampal neurogenesis, improves cognition and resilience^16,29,43^. Given the differential response observed between the rats in the WM paradigm, our findings indicate that the experience of the stressor induced by the LH paradigm may indeed vary between the individual animals as well as have a varying impact on coping abilities over time. The exact reason why some individuals learn to cope, while others fail to do so following the same type of stressor, still needs to be explored. Further studies are also needed to access whether the indication of resilience as seen in the WM paradigm, is a consequence of increased attention, improved performance strategies or other.

The differential findings indicate the presence of individual resilience and the development of coping strategies following the exposure to an aversive situation. Here, findings point toward a stress-mediated response. Altogether, stress may in some have a beneficial effect, yet for those individuals unable to tackle these aversive events, consecutive unpleasant episodes may lead to worse cognitive performance later in life.

## Data Availability

The data that support the findings of this study are available from the corresponding author upon reasonable request.
